# Short-term effects of intragastric balloon in association with conservative therapy on weight loss: a meta-analysis

**DOI:** 10.1186/s12967-015-0607-9

**Published:** 2015-07-29

**Authors:** Yiyuan Zheng, Miao Wang, Songhua He, Guang Ji

**Affiliations:** Department of Internal Medicine of Traditional Chinese Medicine, Longhua Hospital, Shanghai University of Traditional Chinese Medicine, Shanghai, 200032 China; Institute of Digestive Diseases, Longhua Hospital, Shanghai University of Traditional Chinese Medicine, Shanghai, 200032 China

**Keywords:** Obesity, Intragastric balloon, Meta-analysis, Efficacy, Safety

## Abstract

Obesity is an epidemic chronic disease which is a risk factor for a number of serious medical conditions. Intragastric balloon, as an alternative, non-surgical treatment approach for the management of obesity, was rejected in previous evidence-based reviews. The object of this review is updating the data and confirming the safety and efficacy of the procedure. In this review, systematic literature retrieve of MEDLINE, EMBASE, CENTRAL and other information sources was performed from inception to December 2014. The quality of selected studies was assessed and meta-analyses of weighted mean differences were made using the inverse variance method. Meta-analyses presented significant effect sizes of −8.9 kg, −3.1 kg/m^2^ and −21.0% for SMG as well as of −1.5 kg and −1.2 kg/m^2^ for LSG, favoring the intervention group. Safety analysis showed that minor complications occurred at a high rate in intervention group, however, no serious or fatal complication was reported in these studies. In conclusion, the current review presents that short-term efficacy for 6 months treatment of intragastric balloon in association with conservative therapy is clinically significant.

**Electronic supplementary material:**

The online version of this article (doi:10.1186/s12967-015-0607-9) contains supplementary material, which is available to authorized users.

## Background

Obesity is a chronic disease which is a risk factor for a number of serious medical conditions such as diabetes mellitus, cardiovascular diseases, cancers, and some other obesity-related diseases [1] and leads to considerable morbidity, substantial mortality and impaired quality of life [2–4]. The World Health Organization’s (WHO) latest projection indicated that there were more than 1.9 billion overweight adults and at least 600 million people who were obese in 2014. And by 2015, the numbers would be approximately 2.3 billion and 700 million respectively [5].

According to current guidelines, options for treatment include behavioral modification (which include physical exercise, dietary modification, caloric restriction and psychosocial interventions), pharmacotherapy (such as orlistat), and bariatric surgery. As systematic reviews [6, 7] have shown that it is the most effective way to achieve sustained weight reduction, bariatric surgeries are suggested for the patients who failed in non-surgical interventions and who have severe obesity or moderate obesity with a major obesity-related comorbidity [8]. However, as the substantial co-morbidities, serious adverse effects and high surgical risks, there is a part of obese objects (especially adolescents, who have been more attention paid in recent years) who does not qualify for, or does not give consent to, the bariatric procedure.

As a minimally invasive procedure inducing weight loss by reducing the gastric reservoir capacity, leading to premature satiation and prolonged satiety, and regulating hormone mediated signal transduction, intragastric balloon (IGB) insertion performs an alternative, non-surgical treatment approach for the management of obesity in those individuals who refuse or are unsuitable for bariatric surgeries.

The procedure has first been performed in the early 1980s [9] as one of the earliest devices used for endoscopic bariatric intervention and continued to become a worldwide epidemic in recent years. Some systematic reviews [10, 11] have been published during the period, however, different results were turned out which leads to a confusing conclusion.

In these two reviews, the early one [10] which obtained a positive consequence used a quite broad inclusion criteria. Plenty of cohort studies and just two controlled trials were included in this review which means the validity of this research could be limited in somehow. And the later one [11], which used a rigorous inclusion criteria and performed a series of quality assessments, turned out a negative consequence as the small numbers of trials within any component comparisons.

However, both of these two reviews were accomplished several years ago. In recent years, ongoing innovations have resulted in newer designs and placement techniques which improve the efficacy as well as reduce adverse reactions. Hence, with a growing body of evidence supporting the safety and short-term efficacy of the procedure, we decided to make an update to confirm it as well as made a review of the current status of Intragastric Balloon to increase knowledge, foster research, and promote better treatment for people with obesity and their loved ones.

## Methods

### Search strategy and study selection

An electronic literature search was performed on OVID with the following databases: MEDLINE, EMBASE, CENTRAL. The search strategy followed the identification and screening guidelines established by the Preferred Reporting Items for Systematic Reviews and Meta-Analyses (PRISMA) statement [12] without language and publication restrictions was presented in Additional file 1: Appendix. The search was supplemented by screening other databases such as Center Watch, Clinical Trials and Current Controlled Trials. Manual retrieval of the reference lists of selected papers and relevant systematic reviews complemented the electronic search.

The inclusion criteria were summarized as randomized, parallel controlled clinical trials comparing the efficacy and safety of intragastric balloon with conservative therapy were potentially eligible. The updated articles were selected when double or serial publications were found by the same research group to avoid double counting objects. Trials satisfied the inclusion criteria were also excluded as there is no available data. Each record was independently assessed by two researchers and conflicts resolved by a third investigator.

### Data extraction and quality assessment

A standard data extraction method was conformed in each trial to record the following properties: study characteristics (including year of publication, country and sample size); demographic and anthropometric measures (age, gender and BMI); intervention therapy; comparison therapy and outcomes. Outcomes include efficacy indicators [weight loss (WL), BMI and excess weight loss (EWL)] and safety indicators (abdominal pain, flatulence, nausea, vomiting, gastric erosion and gastric ulcer) were extracted at the time points of end-of-treatment for which data were available as well as at the post closest time points for which were not. Data were combined using weighted means and standard deviations as intervention group versus comparison group while there is more than one controlled parallel-arm in a trial. Each trial was independently extracted by two reviewers and checked each other for accuracy.

The quality of trials was assessed using the following evidence-based parameters: randomization technique, blinding design, method of allocation concealment, descriptions of attrition, preliminary analysis, intention-to-treat (ITT) analysis and report of adverse events. Finally, we evaluated a Jadad scale score [13] to assess the risk of study bias. However, the score was not used as a criterion for selection of trials, whereas some parameters were used only for descriptive purposes. Two reviewers independently assessed each study. Disagreements were resolved by a third one.

### Data synthesis and analysis

Data manipulation and analysis were performed using Comprehensive Meta-Analysis Version 2 in conjunction with Excel 2013 [14].

Results were calculated by weighted mean differences (WMDs) and associated 95% confidence intervals (CIs). Subgroup analysis were performed, grouping by the treatment time whether less than six months or not as less than six months group (LSG) and six months group (SMG) to explore the relationship between efficacy and treatment time. For adverse events, we just reported the incidences of adverse events occurred during the period of treatment. No meta-analysis was performed for low comparability as sham procedure was not performed in most comparison groups. Mixed effects analysis models were applied throughout which means random effects models were used to combine studies within each subgroup and fixed effects models were used to combine subgroups and yield the overall effects.

Heterogeneities were estimated using the I^2^ statistics, which indicated the per cent of total variations (within- and between-study) due to between-study variations. I^2^ < 33.3% was considered as an indication of low heterogeneity, 33.3% ≤ I^2^ < 66.7% was considered as an indication of medium heterogeneity, and I^2^ ≥ 66.7% was considered as an indication of high heterogeneity.

Publication biases were explored by using the Begg and Mazumdar adjusted rank correlation test as well as by using the classic fail-safe N method. One study removed analyses were performed to evaluate sensitivities of effects. Funnel plots and meta-regressions were not performed due to small numbers of trials within any component comparisons.

## Results

### Study characteristics

**Fig. 1:**
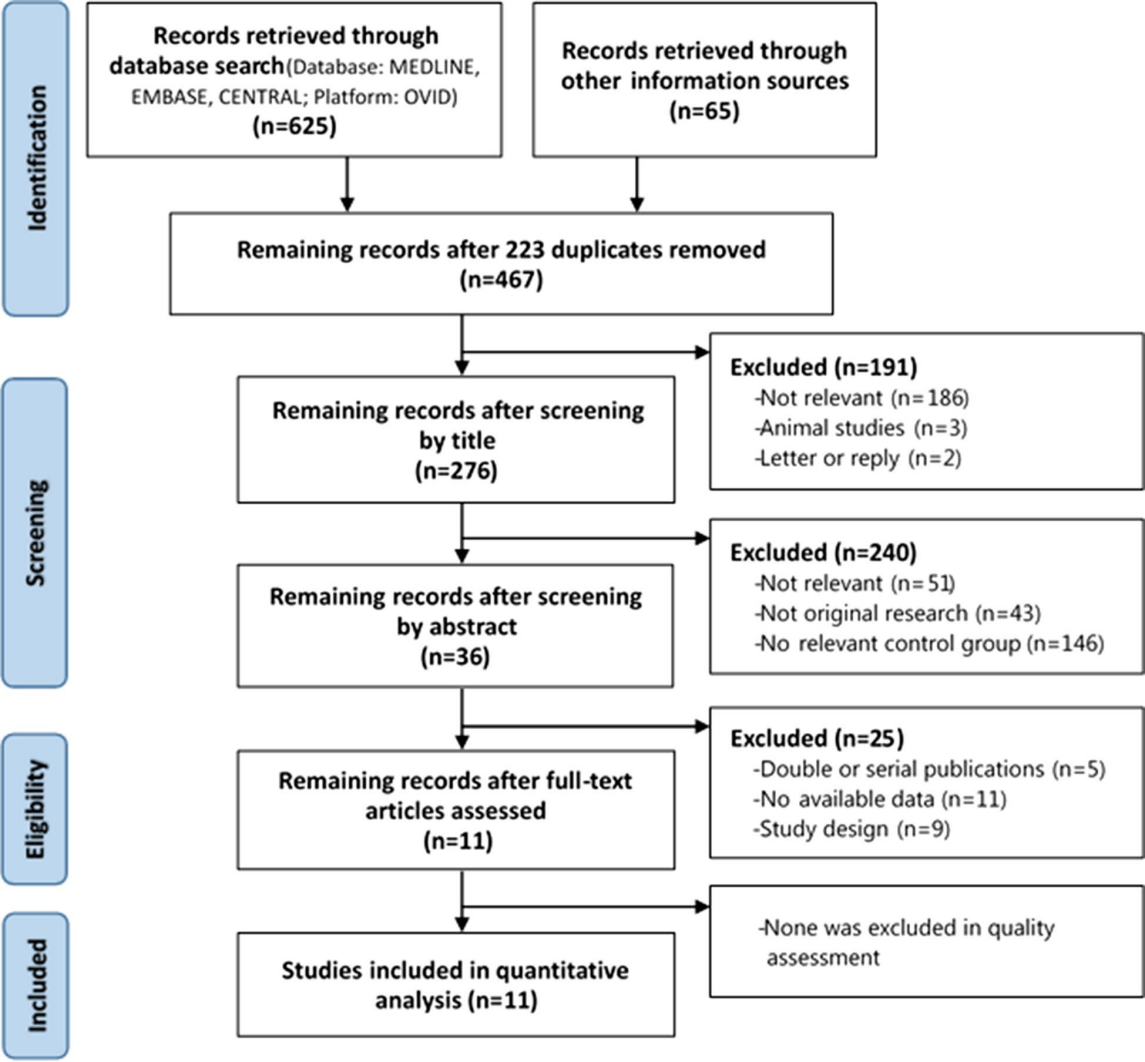
PRISMA flowchart for the selection of studies. Outcomes of the systematic review of the literature by record identification, screening, and analysis in the Preferred Reporting Items for Systematic Reviews and Meta-Analyses (PRISMA) statement flow diagram.

The results of the literature retrieve and selection process are presented in Fig. 1. A total of 690 records were identified, in which 625 were retrieved through electronic database and 65 were supplied by other information sources. 223 duplicates were removed. After screening by title and abstract, we read the full-text of the remaining 36 articles. Nine were rejected as cross-over studies of three as well as not RCT designs of 6 and 11 were excluded because there’s no available data. As removement of five double or serial publications, a final of 11 studies [15–25] were included for quantitative analysis.

Characteristics of the 11 included studies are described in Table 1. Comparative publications were published between 1987 and 2014. Sample size ranged from 22 to 128 (median 36), the mean age for which data were available ranged from 35 to 46 years (median 42 years) and the baseline average BMI ranged from 35.0 to 50.4 kg/m^2^ (median 41.8 and 43.1 kg/m^2^). Of the 11 trials, nine compared intragastric balloon in association with conservative therapy (behavioral modification in all of them and pharmacotherapy plus in just one object) and conservative therapy only, and the other three compared intragastric balloon and observation without any treatment. Five blind studies got sham procedures in comparison group and the others didn’t. The numbers of trials divided into LSG and SMG were 5 and 6 respectively. Finally, quality assessment indicating potential risk for bias is shown in Table 2.

**Table 1:** Characteristics of the 11 included studies

References	Country	Sample size, n	Age, years	Men, %	BMI, kg/m^2^	Intervention therapy	Comparison therapy	Time point, months
Lindor et al. (1987) [15]	USA	22	25–51	9	>30	IGB	Sham	3
Ramhamadany et al. (1989) [16]	England	24	NA	0	41	IGB + BM	Sham + BM	3
Geliebter et al. (1991) [17]	USA	86	NA	19	>40	IGB ± BM	BM/NT	3
Martinez-Brocca et al. (2007) [18]	Spain	22	36 ± 10	23	50.4 ± 7.8	IGB	Sham	4
Konopko-Zubrzycka et al. (2009) [19]	Poland	36	42 ± 12	47	47.2 ± 5.5	IGB + BM	BM	6
Farina et al. (2012) [20]	Italy	50	35 ± 1	22	41.8 ± 0.8	IGB + BM + PT	BM + PT	12
Lee et al. (2012) [21]	Singapore	21	21–65	61	31.5 ± 4.5	IGB + BM	Sham + BM	6
Fuller et al. (2013) [22]	Australia	66	46 ± 9	33	36.4 ± 2.6	IGB + BM	BM	6
Ponce et al. (2013) [23]	USA	30	41 ± 9	13	35.0 ± 2.6	IGB + BM	BM	6
Mathus-Vliegen and Eichenberger (2014) [24]	The Netherlands	40	42 ± 11	10	43.1 ± 6.3	IGB	Sham	3
Mohammed et al. (2014) [25]	Egypt	128	44 ± 9	58	47.7 ± 1.1	IGB + BM	BM	6

*BMI* body mass index, *IGB* intragastric balloon, *NA* not available, *BM* behavioral modification, *NT* no treatment, *PT* pharmacotherapy.

**Table 2:** Quality assessment of included studies

References	Randomization	Allocation concealment	Blinding	Attrition described	preliminary analysis	ITT analysis	Adverse events report	Jadad score
Lindor et al. (1987) [15]	Yes	Unclear	Yes	Yes	Yes	No	Yes	4
Ramhamadany et al. (1989) [16]	Yes	Yes	Yes	Yes	Yes	Yes	Yes	5
Geliebter et al. (1991) [17]	Yes	Unclear	No	Yes	Yes	Yes	Yes	2
Martinez-Brocca et al. (2007) [18]	Yes	Unclear	Yes	Yes	Yes	No	Yes	5
Konopko-Zubrzycka (2009) [19]	Yes	Unclear	No	Yes	Yes	Yes	Yes	2
Farina et al. (2012) [20]	Yes	Unclear	No	Yes	Yes	No	Yes	2
Lee et al. (2012) [21]	Yes	Yes	Yes	Yes	Yes	No	No	4
Fuller et al. (2013) [22]	Yes	Yes	No	Yes	Yes	Yes	Yes	3
Ponce et al. (2013) [23]	Yes	No	No	Yes	Yes	Yes	Yes	3
Mathus-Vliegen and Eichenberger (2014) [24]	Yes	Yes	Yes	Yes	Yes	Yes	No	4
Mohammed et al. (2014) [25]	Yes	Unclear	No	Yes	Yes	Unclear	Yes	2

### Efficacy

#### Weight loss (WL)

**Fig. 2:**
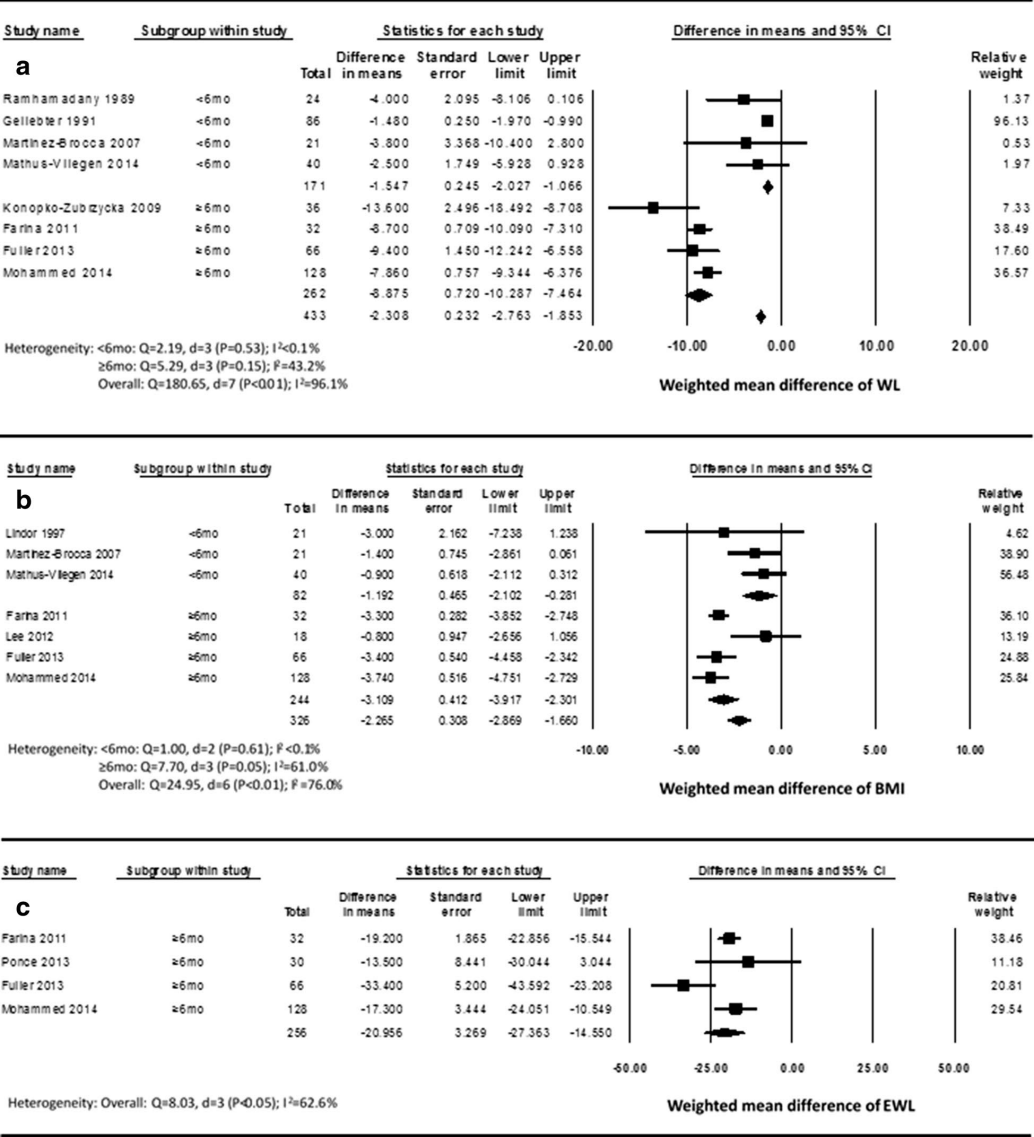
Forest plots for effects of intragastric balloon in association with conservative therapy. **a** Forest plot for SMD of WL in intervention group compared with comparison group. **b** Forest plot for SMD of BMI in intervention group compared with comparison group. **c** Forest plot for SMD of EWL in intervention group compared with comparison group.

Figure 2a presents the effect sizes of eight trials for which information are available on WL. The WMD comparing intervention group and comparison group was −1.5 kg [(−2.0, −1.1), p < 0.01] for LSG, favoring the intervention group; whereas, the corresponding WMD for SMG was −8.9 kg [(−10.3, −7.5), p < 0.01].

Within-group analysis indicated low heterogeneity (I^2^ < 0.1) in LSG as well as medium heterogeneity (I^2^ = 43.3) in SMG. Significant heterogeneity (p < 0.01) was found between LSG and SMG.

No publication bias was found using the Begg and Mazumdar adjusted rank correlation test (p = 0.54) as well as using the classic fail-safe N method (n = 519). One study removed analysis showed an overall WMD of −3.0 kg with stable results (WMDs ranged between −2.4 and −3.0) of seven studies except Geliebter (1991) (WMD = −7.9 kg) using fixed model. However, the corresponding analysis showed an overall WMD of −6.4 kg with stable results (WMDs ranged between −5.5 and −7.4) of all the eight studies using random model.

#### BMI

Figure 2b presents the effect sizes of seven trials for which information are available on BMI. The WMDs comparing intervention group and comparison group were −1.2 kg/m^2^ [(−2.1, −0.3), p = 0.01] and −3.1 kg/m^2^ [(−3.9, −2.3), p < 0.01] for LSG and SMG respectively, favoring the intervention group.

Within-group analysis indicated low heterogeneity (I^2^ < 0.1) in LSG as well as medium heterogeneity (I^2^ = 61.0) in SMG. Significant heterogeneity (p < 0.01) was found between LSG and SMG.

No publication bias was found using the Begg and Mazumdar adjusted rank correlation test (p = 0.13) as well as using the classic fail-safe N method (n = 241). One study removed analysis showed overall WMDs of −2.9 and −2.4 kg/m^2^ with stable results (WMDs ranged between −2.5 and −3.1 as well as between −2.2 and −2.8) using fixed model and random model respectively.

#### Excess weight loss (EWL)

Figure 2c presents the effect sizes of four trials for which information are available on EWL. None of them got treatment less than 6 months. The WMD comparing intervention group and comparison group were −21.0% [(−27.4, −14.6), p = 0.01], favoring the intervention group.

No significant heterogeneity (p < 0.05) was found and medium heterogeneity (I^2^ = 62.6) was assessed according to the pre-specified I^2^ value.

No publication bias was found using the Begg and Mazumdar adjusted rank correlation test (p = 1.00) as well as using the classic fail-safe N method (n = 138). One study removed analysis showed overall WMDs of −19.9 and −21.0% with stable results (WMDs ranged between −18.6 and −21.3 as well as between −18.6 and −22.6) using fixed model and random model respectively.

### Safety

**Fig. 3:**
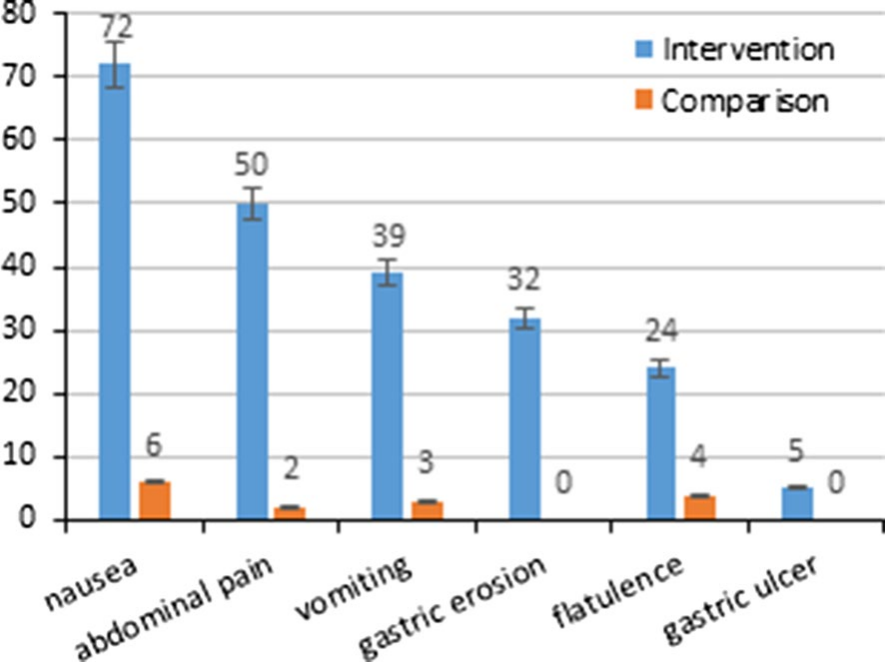
The incidences (%) of adverse events occured during the period of treatment. The adverse events included three major complications (nausea, abdominal pain and vomiting) and three minor complications (gastric erosion, flatulence and gastric ulcer).

As safety index, the incidences of adverse events occurred during the period of treatment are presented in Fig. 3. Of the 11 included comparative studies, 8 reported the relevant information which included 6 complications (nausea, abdominal pain, vomiting, gastric erosion, flatulence and gastric ulcer). For nausea, 122 events occurred in a total of 169 objects (72%) in intervention group; whereas, the corresponding number was 7 of 115 (6%) for comparison group. 80 of 159 (50%) and 2 of 117 (2%) were reported in intervention and comparison group respectively for abdominal pain as well as 78 of 202 (39%) and 4 of 160 (3%) for vomiting. Complications were reported as 27 of 85 (32%), 31 of 127 (24%) and 7 of 138 (5%) for gastric erosion, flatulence and gastric ulcer in intervention group, respectively. In comparison group, no gastric erosion or ulcer was found and flatulence happened in four individuals of 91 objects (4%). One brief hypoxia during device removal was reported in Ponce (2013) and no other fatal event such as gastric-intestinal perforation or intestinal obstruction was reported in these studies.

## Discussion

The current review systematically identified and assessed a wide range of evidence regarding the efficacy and safety of intragastric balloon in association with conservative therapy versus conservative therapy only. As the initial post balloon placement effect (such as significant nausea and vomiting) would result in immediate self-unmasking for those patients randomized to the treatment group, most trials didn’t perform blind design. No-blinding studies leading to low quality assessment were not excluded because the small number of available trials.

Meta-analyses presented significant effect sizes of −8.9 kg, −3.1 kg/m^2^ and −21.0% for SMG as well as of −1.5 kg and −1.2 kg/m^2^ for LSG, favoring the intervention group. As a modest weight loss of 5–10% is associated with clinically significant benefits reducing the risk of diabetes, hyperlipemia, hypertension and associated cardiovascular diseases [26–28], standards were confirmed as −5 kg, −2 kg/m^2^ and −15% for modest efficacy as well as −10 kg, −4 kg/m^2^ and −25% for obvious efficacy. According to these standards, modest efficacy for intragastric balloon as a conjunction therapy to conservative therapy was achieved in SMG. As conservative treatments were performed in most individuals of comparison groups, clinically significant benefits for 6 months treatment of intragastric balloon in association with conservative therapy could be confirmed conservatively. However, no clinically significant benefit was found in LSG which was likely due to the small number of trials and the heterogeneity of the studies in each group.

Subgroup analyses indicated that heterogeneity between long-term and short-term treatment was exactly significant. Combined with previous analyses, the efficacy of long-term treatment presented a superiority to short-term treatment. These results suggested that for improved efficacy, balloon treatment might need to be longer according to the individual’s gastric tolerance. However, as researches reported long-term placement was associated with a trend towards greater procedure- and device-related complications, the treatment time should not be much longer than 6 months.

High heterogeneity was found as most was due to between-group differences. Within-group analysis showed low heterogeneity in LSG as well as medium heterogeneity in SMG. However, attention should still be paid as the validity of tests was limited with small number of trials in each analysis. Differences in material, volume of balloons, patient characteristics, and especially in treatment of conservative therapy were potential sources for heterogeneity. Analyses were not performed for the same reason as small numbers of trials.

No publication bias was found using the Begg and Mazumdar adjusted rank correlation test as well as using the classic fail-safe N method in all analyses which indicated a low risk of publication bias could be believed. It’s also an indicator of stable that much bigger classic fail-safe N than the number of included studies. Sensitivity analyses showed stable results on the other hand except analysis of WL with Geliebter (1991) using fixed model, however, the corresponding analysis using random model was stable. Study was re-read and the reason was considered as rigorously controlled intervention inducing small variance in each group which resulted in a big weight and the combination of data went a step further on this way. However, the effect size excluded Geliebter (1991) was better than which included it and was still clinically significant.

Safety analysis showed that complications occurred at a high rate in intervention group. Fortunately, the complications were all minor and most of them were self-healing reactions which would disappear after a few days. In the meaning time, some researches [29–31] were committed to improving these conditions by developing new balloons or new placement techniques as well as by associating with other treatment such as antiemetic. Although some serious complications such as gastric-intestinal perforation or intestinal obstruction were not reported in this review, it should be noted that there were some trials meeting these reactions [10, 32, 33].

Compared with the previous systematic reviews [10, 11], a clinically significant benefit was verified which was due to a sufficient number of available studies. Inclusion criteria was rigorously designed, some previous included controlled trials were also excluded as the cross-over design studies used different program designs which might bring unstable results. Another important superiority is that subgroup analysis for treatment time was performed in this review. It was also confirmed that the efficacy of long-term treatment presented a superiority to short-term treatment which was not disclosed in previous. In consideration of the complications, this research made a suggestion that for improved efficacy, the time of balloon treatment might need to be longer according to the individual’s gastric tolerance but should not be longer than 6 months.

Limitations should be acknowledged that the number of trials for subgroup analysis was small in each group and heterogeneity of the studies such as the models of balloons and the different conservative treatments was not analyzed. The most important is that analyses were just performed at the time points of end-of-treatment. Most long-term follow up studies were cohort studies and the randomized parallel controlled clinical trials were too less to analyze. What’s more, the efficacies of maintenance for long-term follow up were quite different in these trials. Some [34–38] reported that the treatment induced a successful weight loss and maintenance, a better control of comorbidities and a better quality of life. However, others [39–41] reported that most patients lost the reduction after balloons were removed and the long-term results were poor. According to studies [42–44] exploring the relationship between efficacy and various factors, the difference was considered as a result inducing by a series of factors such as compliance with long-term behavior modification, complications, and mental state.

In recent years, new balloons such as adjustable balloon, swallowable balloon, self-emptying balloon and dual intragastric balloon (two connected balloons) were tested for improving effect or reducing complications [29, 30, 45, 46]. Repeated treatment with balloons was also researched, however, the outcomes were confused [47, 48]. A part of researches [49, 50] suggested that the procedure should be associated with some new methods such as psychotherapy and even endoscopic gastrointestinal bypass surgery (such as EndoBarrier gastrointestinal liner). Others [51–53] declared that intragastric balloon could be an approach smoothing the path to bariatric surgery.

In conclusion, as an alternative, non-surgical treatment approach for the management of obesity in those individuals who refuse or are unsuitable for bariatric surgeries, the short-term efficacy has been verified in this review. However, more new designed, rigorously controlled and randomized long-term follow-up studies were needed to assess long-term efficacy and safety of the procedure.

## Conclusions

The current review summarizes a wide range of evidence regarding the efficacy and safety of intragastric balloon in association with conservative therapy on weight loss in obese objects. Short-term efficacy for 6 months treatment of intragastric balloon in association with conservative therapy is clinically significant.

## Additional file

Additional file 1: Appendix. Search strategy. And the data presented the search strategy in this review.

## Abbreviations

IGB: intragastric balloon
WL: weight loss
EWL: excess weight loss
WMD: weighted mean difference
CI: confidence interval
LSG: less than six months group
SMG: six months group
PRISMA: Preferred Reporting Items for Systematic Reviews and Meta-Analyses
