# Supplementary material for: Short-term effects of intragastric balloon in association with conservative therapy on weight loss: a meta-analysis
Source: J Transl Med. 2015 Jul 29;13:246. doi: 10.1186/s12967-015-0607-9 (PMC4517653; doi:10.1186/s12967-015-0607-9)
Supplement: Additional file 1: Appendix. — Search strategy. And the data presented the search strategy in this review. [file 12967_2015_607_MOESM1_ESM.doc]

**Appendix**: Search strategy

| Search strategy(Database: MEDLINE, EMBASE, CENTRAL; Platform: OVID; Date: 1946-2014.9) |
| --- |
| 1 obesity.sh. 408300 |
| 2 overweight.sh. 14183 |
| 3 body weight.sh. 383480 |
| 4 abdominal fat.sh. 5067 |
| 5 body mass index.sh. 90743 |
| 6 weight loss.sh. 28750 |
| 7 fat$.af. 1899636 |
| 8 adipos$.af. 231749 |
| 9 obes$.af. 610460 |
| 10 overweight.af. 105645 |
| 11 over weight.af. 5920 |
| 12 body weight.af. 623810 |
| 13 body mass ind$.af. 304693 |
| 14 BMI.af. 235663 |
| 15 EWL.af. 2637 |
| 16 weight reduc$.af. 122956 |
| 17 weight los$.af. 159767 |
| 18 weight decreas$.af. 6202 |
| 19 weight control$.af. 15049 |
| 20 or/1-19 3157933 |
|  |
| 21 gastric balloon.sh. 661 |
| 22 gastric balloon$.af. 1116 |
| 23 gastric bubble$.af. 137 |
| 24 intragastric balloon$.af. 1020 |
| 25 intragastric bubble$.af. 9 |
| 26 stomach balloon$.af. 28 |
| 27 stomach bubble$.af. 88 |
| 28 BIB.af. 884 |
| 29 IGB.af. 1059 |
| 30 or/21-29 3465 |
|  |
| 31 randomized controlled trial.pt. 737532 |
| 32 controlled clinical trial.pt. 174365 |
| 33 clinical trial.pt. 772878 |
| 34 randomized controlled trial.sh. 742216 |
| 35 controlled clinical trial.sh. 477466 |
| 36 clinical trial.sh. 1338765 |
| 37 research design.sh. 84690 |
| 38 random allocation.sh. 102697 |
| 39 double blind method.sh. 235223 |
| 40 single blind method.sh. 32675 |
| 41 placebos.sh. 55164 |
| 42 comparative study.sh. 2360835 |
| 43 prospective studies.sh. 448127 |
| 44 evaluation studies.sh. 200938 |
| 45 follow up studies.sh. 552172 |
| 46 random$.ti,ab. 2058827 |
| 47 alloc$.ti,ab. 197441 |
| 48 assign$.ti,ab. 542514 |
| 49 design$.ti,ab. 2661527 |
| 50 clin$ adj3 trial$.ti,ab. 616979 |
| 51 (singl$ or doubl$ or tripl$ or trebl$) adj3 (blind$ or mask$).ti,ab. 451084 |
| 52 placebo$.ti,ab. 514776 |
| 53 control$.ti,ab. 6383511 |
| 54 prospective$.ti,ab. 1163045 |
| 55 or/31-54 13216717 |
| 56 animals.sh. 5428801 |
| 57 animal experimentation.sh. 2718 |
| 58 models animal.sh. 32368 |
| 59 humans.sh. 14238736 |
| 60 or/56-58 5429264 |
| 61 60 not 59 3919878 |
| 62 55 not 61 12036919 |
| 63 20 and 30 and 62 625 |
